# Osteo-Arthritis

**Published:** 1907-12-07

**Authors:** 


### OSTEO-ARTHRITIS.

Osteo-arthritis is a common and a troublesome complaint. Its method of causation has given rise to an infinite amount of speculation; but in spite of this it can hardly be claimed that we have any definite knowledge on the subject. Various opinions are held on the question whether heredity plays any part in its production. Statistics on this point are difficult to collect accurately; firstly, because the disease has been saddled by writers on medicine and surgery with a great number of different names, and secondly because among the poorer classes, among whom this condition is so prevalent, very little accurate information can be obtained about the patient's family history. It appears that the disease is more common in women than it is in men, and that the age at which it most often makes its appearance is the period between the fortieth and fiftieth years. It is interesting to note that the chronic polyarticular variety which attacks the joints of the hands is more common in females, while in males the chronic monarticular form which affects the larger articulations of the limbs is more frequently met with.

In a great number of cases a definite history of antecedent injury to the joint can be traced. In some the injury is a more or less sudden or violent one, as, for instance, a twist of the knee at football or a definite sprain. In others no definite force has been applied to the joint, but owing to the patient's occupation it has been subjected to constant overuse. Osteo-arthritis in the wrists in gardeners is attributable to this cause as the result of constantly digging; while in labourers who are accustomed to carry loads of bricks in a hod on their shoulder, the shoulder-joint is frequently affected. Exposure to wet or cold seems to have a definite relation to the condition, and here again the exposure may be a single event, such as getting wet through or sleeping in a damp bed, or it may be the constant exposure to which the patient's occupation renders him liable. Sailors and fishermen, for instance, are said to suffer from osteo-arthritis of the knees from this cause. In recent years the opinion has gained ground that the condition is either the result of a definite infection by a micro-organism which is specific, or is a sequela of some of the more ordinary acute infections. Whether in either case the joint is disorganised by the direct action of the micro-organism itself, or degenerate as the result of toxins manufactured elsewhere and distributed by the circulatory system, there is no evidence. The fact that osteo-arthritis when it occurs in children is often associated with enlargement of the lymphatic glands, for which no other cause can be found, is certainly suggestive of a bacterial origin. Most of the acute zymotic diseases have at one time or another been held responsible for the production of osteo-arthritis. Further than this it has been suggested that disturbances of the nervous system, whether central or peripheral, can be said to account for its onset. By central disturbances one means such conditions as prolonged worry or mental strain, which frequently precede an attack. Derangement of the peripheral nervous system on the other hand more often occurs during the course of the disease; peripheral neuritis and trophic lesions are found in the limb which contains the affected joint. Finally, it may be said that almost any disease which profoundly affects the patient's general health may lower his resistance and predispose to an attack of osteo-arthritis. But it will be seen that, although any number of hypotheses are to be found, conclusive facts are far to seek. In the writer's experience it is more common to find a history of antecedent injury or exposure than of any other of the conditions mentioned. But it must be remembered that almost any patient with sufficient encouragement can recall a past injury to a given joint, and all such statements must be accepted with caution. It is only fair to say that although many patients will give such a history, very few of them bear such surgical evidence of severe damage to the joint or the part as to put the matter beyond dispute. It seems reasonable to continue our inquiries into the causation of this disease along bacteriological lines; if only for the reason that so many diseases whose ætiology was a mystery before, became perfectly intelligible as soon as a bacterial cause for them was found.

### Pathology.

The pathological changes found in the affected joints present several different aspects; whether these varieties can be classified so as to correspond to a constant mode of causation in each case is doubtful. Goldthwait of America has examined a large number of osteo-arthritic joints by means of *x*-rays, and has come to the conclusion that they fall, pathologically speaking, into three main classes. In the first no change can be seen in the bones forming the joint, the synovium and structure external to the joint alone being affected. In the second the cartilage atrophies and the bone tends to undergo rarefaction, while in the third the bone itself is denser than normal, and there is much osteophytic outgrowth. These varieties he calls respectively the infective, the atrophic, and the hypertrophic. It seems difficult to make his results correspond to the acknowledged ideas of the pathology of the disease. Post-mortem examination of such joints, both microscopic and macroscopic, has led us to suppose that the disease almost always starts in the cartilage. The first change is the appearance of small outgrowths on the articular surface, consisting of cartilage cells arranged with their long axes at right angles to the surface. These become ossified; and if they are situated at a place where they are subjected to the friction caused by movement at the joint they are worn away and expose the subjacent bone, which becomes eburnated; if on the other hand they are round the edges of the joint, they persist as osteophytes. Later the synovial membrane becomes thickened and is covered with hypertrophied villous tufts which are fibrous at first but often become calcified. The eburnated surface of the exposed shaft is worn away in grooves, which correspond to the axes of the movements of the joint, while the subjacent cancellous tissue undergoes rarefaction.

*(To be continued.)*

